# Supplementary material for: Genome-wide analysis highlights genetic admixture in exotic germplasm resources of Eucalyptus and unexpected ancestral genomic composition of interspecific hybrids
Source: PLoS One. 2023 Aug 8;18(8):e0289536. doi: 10.1371/journal.pone.0289536 (PMC10409294; doi:10.1371/journal.pone.0289536)
Supplement: S2 File — Results of the four supervised estimators of Puechmaille (2016) to detect the number of clusters implemented in the web server StructureSelector (Li and Liu 2018) indicating that the germplasm set is most likely structured in 18 clusters after modelling with a variable number of k from 2 to 30 using FastStructure. (DOCX) [file pone.0289536.s002.docx]

**Supporting information to:** Oliveira et al. 2023. Genome-wide analysis highlights genetic admixture in exotic germplasm resources of Eucalyptus and unexpected ancestral genomic compositions of interspecific hybrids.

**S1 File.** Results of the four supervised estimators of Puechmaille (2016) implemented in the web server StructureSelector (Li and Liu 2018) indicating that the germplasm set is most likely structured in 18 clusters after modelling with a variable number of k from 2 to 30 using FastStructure.

**
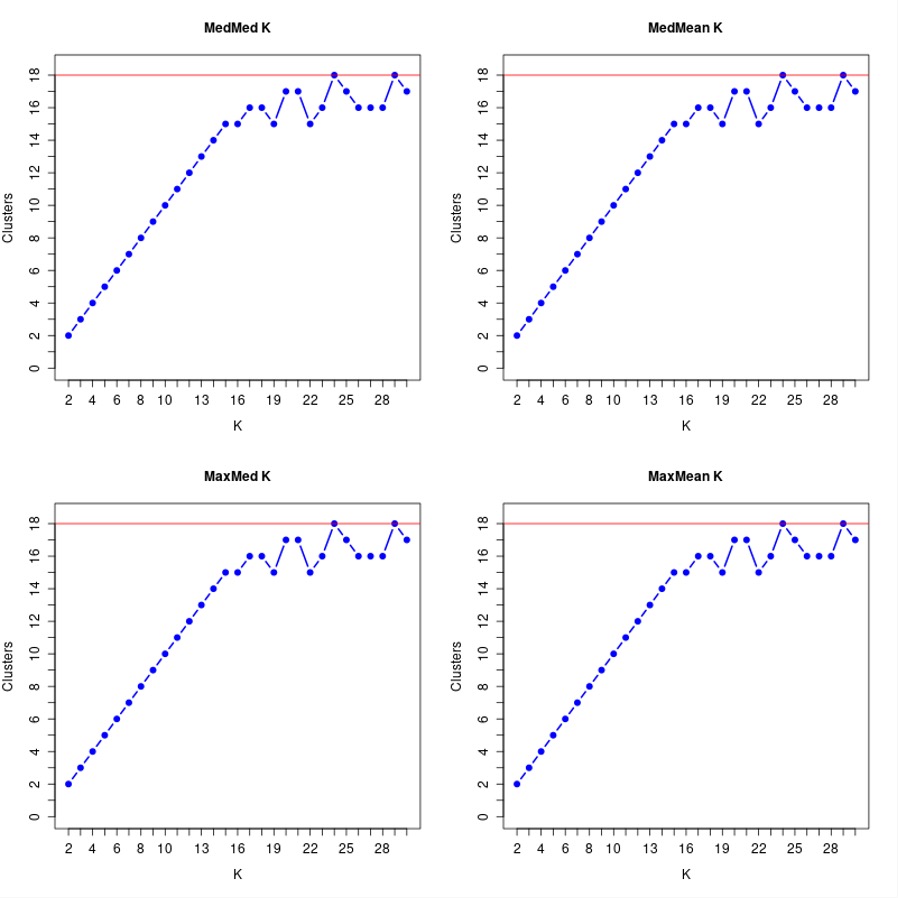
**
