# Supplementary material for: Genome-wide analysis highlights genetic admixture in exotic germplasm resources of Eucalyptus and unexpected ancestral genomic composition of interspecific hybrids
Source: PLoS One. 2023 Aug 8;18(8):e0289536. doi: 10.1371/journal.pone.0289536 (PMC10409294; doi:10.1371/journal.pone.0289536)

**Supporting information to:** Oliveira et al. 2023. Genome-wide analysis highlights genetic admixture in exotic germplasm resources of *Eucalyptus* and unexpected ancestral genomic compositions of interspecific hybrids.

**S2 File.** Population structure analyses of the *Eucalyptus* species and hybrids clustered with variable numbers of clusters (*K*) from 2 to 4 separating the *Eucalyptus* sections (*Maidenaria, Latoangulatae* and *Exsertaria*), while displaying admixture in species of section *Latoangulatae*.


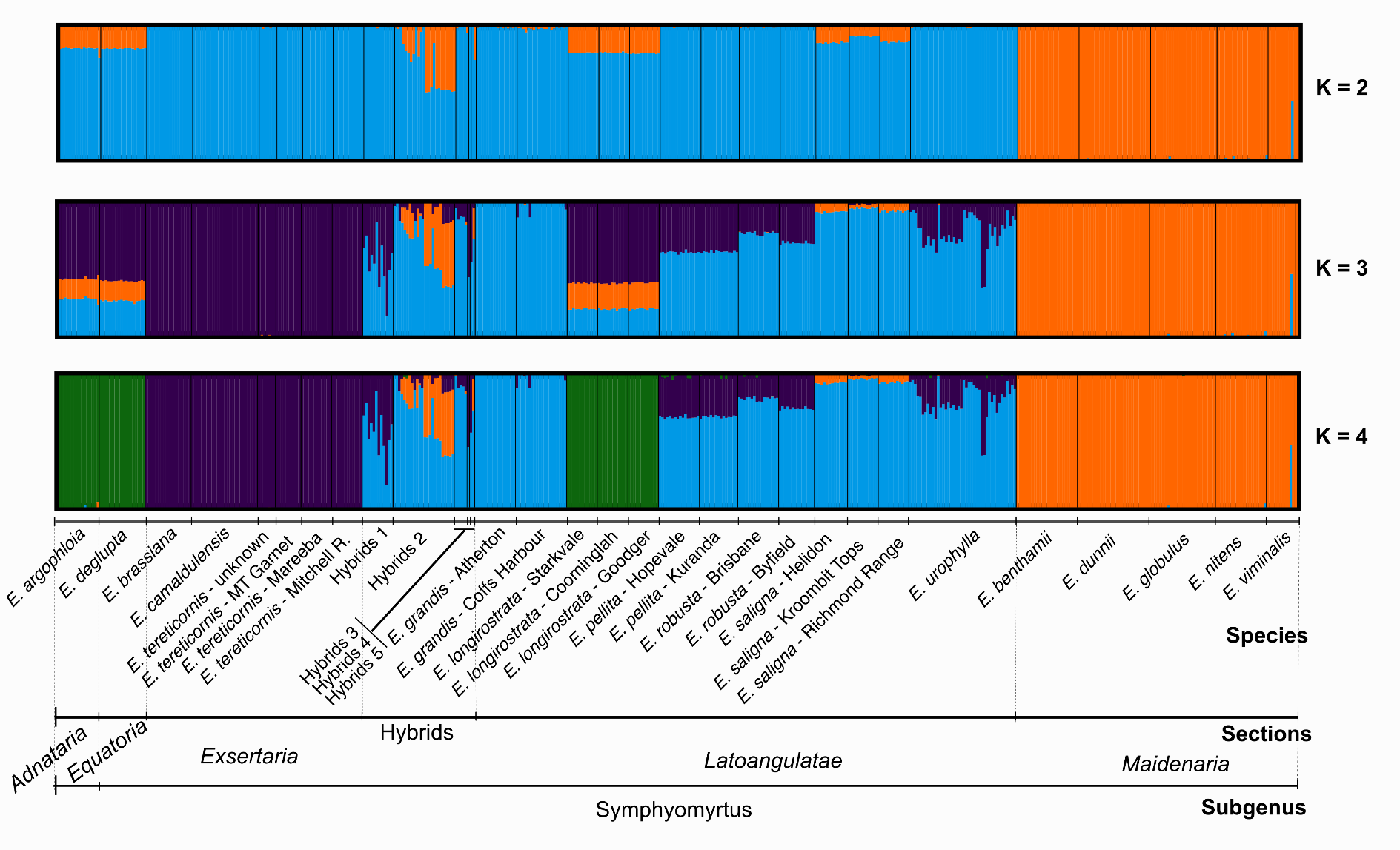

Supplement: S4 File — Population structure analyses of the Eucalyptus species and hybrids clustered with variable numbers of clusters (K) from 2 to 4 separating the Eucalyptus sections (Maidenaria, Latoangulatae e Exsertaria), while displaying admixture in species of section Latoangulatae. (DOCX) [file pone.0289536.s004.docx]
