# Supplementary material for: Genome-wide analysis highlights genetic admixture in exotic germplasm resources of Eucalyptus and unexpected ancestral genomic composition of interspecific hybrids
Source: PLoS One. 2023 Aug 8;18(8):e0289536. doi: 10.1371/journal.pone.0289536 (PMC10409294; doi:10.1371/journal.pone.0289536)

**Supporting information to:** Oliveira et al. 2023. Genome-wide analysis highlights genetic admixture in exotic germplasm resources of *Eucalyptus* and unexpected ancestral genomic compositions of interspecific hybrids.

**S5 File.** Population structure analyses of the *Eucalyptus* species and hybrids clustered with variable numbers of clusters (*K*) from 20 to 30, beyond the most likely model with k=18. The figure indicates that even with more complex models, most provenances within species could not be discriminated when all samples were analyzed together.


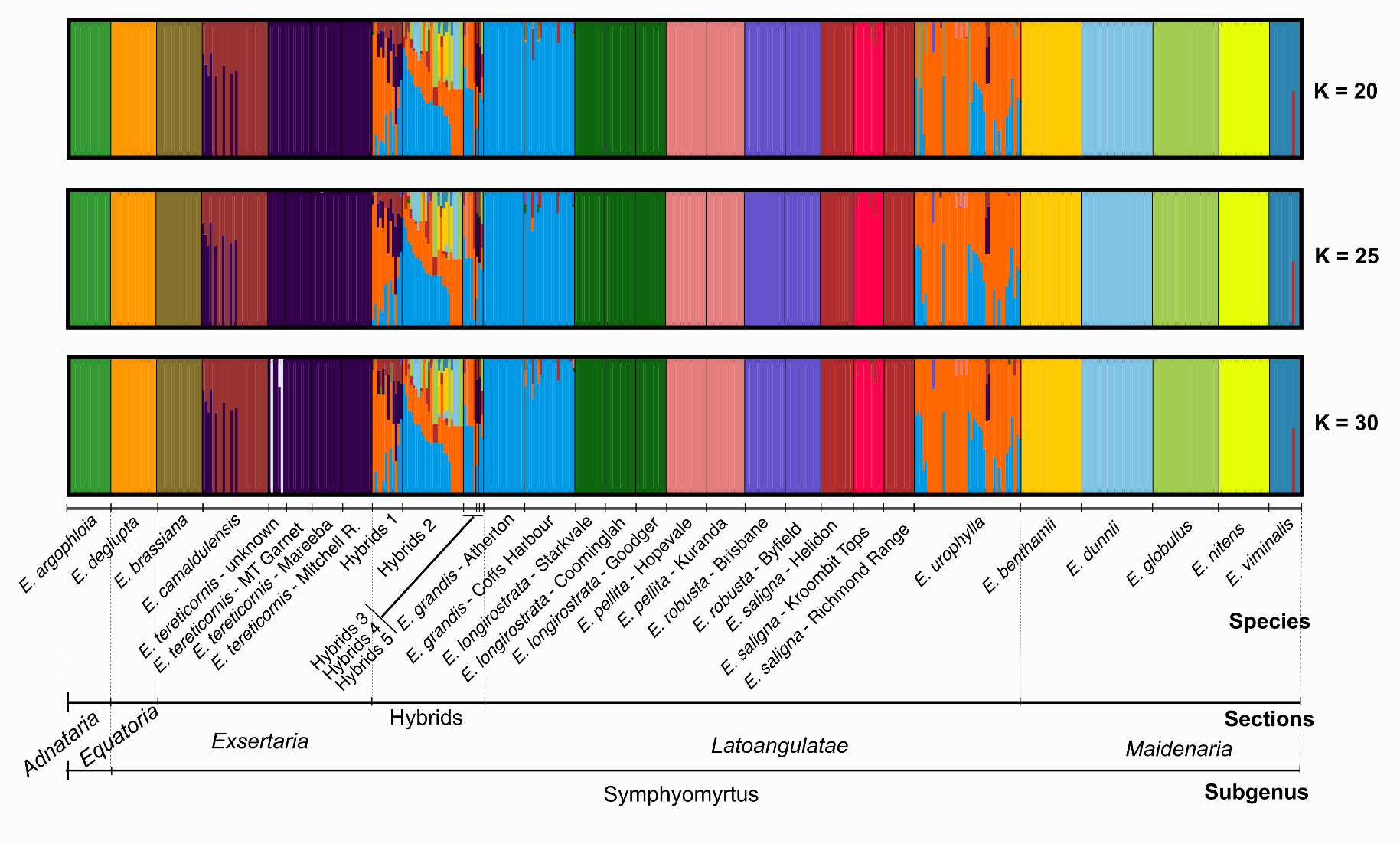

Supplement: S5 File — Population structure analyses of the Eucalyptus species and hybrids clustered with variable numbers of clusters (K) from 20 to 30, beyond the most likely model with K = 18. (DOCX) [file pone.0289536.s005.docx]
