# Supplementary material for: Genome-wide analysis highlights genetic admixture in exotic germplasm resources of Eucalyptus and unexpected ancestral genomic composition of interspecific hybrids
Source: PLoS One. 2023 Aug 8;18(8):e0289536. doi: 10.1371/journal.pone.0289536 (PMC10409294; doi:10.1371/journal.pone.0289536)
Supplement: Attachment — Submitted filename: Responses to the reviewers PONE-D-23-13755.docx [file pone.0289536.s007.docx]

**Response letter for Revised manuscript PONE-D-23-13755 Oliveira et al.**

**Genome-wide analysis highlights genetic admixture in exotic germplasm resources of Eucalyptus and unexpected ancestral genomic compositions of interspecific hybrids**

**Responses to the Editor’s comments**

Editor Comments (in bold) and responses (R:) follow thereafter.

**1. Please ensure that your manuscript meets PLOS ONE's style requirements, including those for file naming.**

R: We have followed closely the PLOS ONE style requirements

**2. We note that Figure 1 in your submission contain [map/satellite] images which may be copyrighted. All PLOS content is published under the Creative Commons Attribution License (CC BY 4.0), which means that the manuscript, images, and Supporting Information files will be freely available online, and any third party is permitted to access, download, copy, distribute, and use these materials in any way, even commercially, with proper attribution. For these reasons, we cannot publish previously copyrighted maps or satellite images created using proprietary data, such as Google software (Google Maps, Street View, and Earth). For more information, see our copyright guidelines:** [**http://journals.plos.org/plosone/s/licenses-and-copyright**](http://journals.plos.org/plosone/s/licenses-and-copyright)**. We require you to either (1) present written permission from the copyright holder to publish these figures specifically under the CC BY 4.0 license, or (2) remove the figures from your submission.** In the figure caption of the copyrighted figure, please include the following text: “Reprinted from [ref] under a CC BY license, with permission from [name of publisher], original copyright [original copyright year].”

R: The base map used in the figure was downloaded from "World boundaries" of the World Bank which is free for use under CC_by_4.0 license. The link where the map was obtained is the following <https://datacatalog.worldbank.org/search/dataset/0038272/World-Bank-Official-Boundaries>. We have improved the text in Material and Methods and figure legend including the details on the source and CC by 4.0 permission of the base map used.

**3. Please review your reference list to ensure that it is complete and correct. If you have cited papers that have been retracted, please include the rationale for doing so in the manuscript text, or remove these references and replace them with relevant current references. Any changes to the reference list should be mentioned in the rebuttal letter that accompanies your revised manuscript. If you need to cite a retracted article, indicate the article’s retracted status in the References list and also include a citation and full reference for the retraction notice.**

R: We have used EndNote 20 to prepare the references using the PlosONE style and further checked the completeness of the references.

**Abstract**

**Line 39, "material" may need a plural form.**

R: Correction made

**Line 44, "composition" may need a plural form.**

R: Correction made

**Introduction**

**The first sentence may be revised to "Eucalyptus is a genus including highly diverse species ruling the forest across a large part** **of Australia and neighboring islands."**

R: I party agreed with the suggestion. Actually, it is correct that the genus is diverse as it encompasses a very large number of species (more than 900). Furthermore, its ecological relevance goes way beyond forests. As many species eucalypts are not trees but shrubs, the genus is a keystone component across the entire forest landscape. So, the sentence now reads: “Eucalyptus is a highly diverse genus of tree species from Australia and neighboring islands ruling the forest landscape across large part of Australia and neighboring islands.

**"in what regards" may be changed to "to".**

R: Change made

**"taxonomic classification" may be changed to "classification" or "taxonomy".**

R: Change made

**Results**

**Table 3, the column "Species (Provenance" may be better viewed with "align left", other than "align center".**

R: Change made

**Line 293, May "composition" be a plural form? This word is used inconsistently in the paper, in terms of plural or singular form, such as in title, line 294, 297, 301, 306, etc. Similar issue exists with the word "distance" in the paper.**

R: Thanks for the observation. The noun “composition” can be plural form. See at: <https://www.collinsdictionary.com/dictionary/english/composition#:~:text=(k%C9%92mp%C9%99z%C9%AA%CA%83,Word%20forms%3A%20plural%20compositions)>

However, specifically in the text, its use is as an uncountable noun and it should actually be singular, unless a specific syntax agreement to plural was necessary. In just one case we used in plural, on line 118 “… or aim at deliberately exploiting provenance and species complementarity by building specific genomic **compositions** by interspecific hybridization”. In this case the plural form would be advised as I am referring to different hybrids that will correspond to different compositions.

**Responses to Reviewer 1 comments**

**In material and methods, please provide the details of location of Eucalyptus in Brazil if possible. Maybe it can be added in Table1.**

R: Actually, eucalypts are planted throughout the entire country in Brazil. We therefore believe that a map of the locations where eucalypts are planted in Brazil would not be relevant to this particular study, as its focus was on the genetic characterization of germplasm, irrespective where it is planted.

Regarding the hybrids studied in this work (Table 1) they are all located in a single location at the experimental station of the Institute for Forestry Research (IPEF) as specified in Material & Methods. We added the geographic coordinates or that experimental station. For the germplasm sources of the pure species, the locations are where the germplasm collection sources are actually planted. These locations are not a relevant information as no phenotypic data is used in the study that could be impacted by the specific environment where they are located. The relevant information for the objective of the study is the original location of the seed sources (provenances) and these are shown in detail in Figure 1.

**In result and discussion, in most of hybrids, higher proportion of E. grandis genome in the hybrid composition was identified. The authors thought the fast growth was one of reason. As we known, why did E. urophylla known as one of fast growth species not happen in the hybrids?**

R: Actually, almost all hybrids also had a significant proportion of E. urophylla genome as can be clearly seen in Figure 4 (red color). Actually, the proportion of E. urophylla was higher than the 50% expected in most of the hybrids in the first group (Hyb01- through Hyb-11) but lower than expected in hybrids Hyb-17 through Hyb-20. See Figure 4 please.

**A large number of sentences were elusive and the quality of the English writing must be improved (example of sentence: L94-97 "However, issues still remain for example for taxa in section Latoangulatae, that still distort the phylogeny due to their intermediate nature when compared to more densely clustered taxa in other sections [8]", and so on.)**

R: We improved this sentence and went through the entire manuscript checking for potential unclear sentences and improved when deemed necessary. Furthermore, we had a couple of native speakers check our manuscript for such occurrences and make suggestions for improvements. We have also rearranged some of the texts in the introduction for a more fluid reading and better connection among the paragraphs.

Thank you for your consideration. We look forward to hearing from you.

Sincerely,

**Dario Grattapaglia**

**Plant Genetics Laboratory, Embrapa - Recursos Genéticos e Biotecnologia**

**e-mail: dario.grattapaglia@embrapa.br**
